# Impact of humid climate on rheumatoid arthritis faecal microbiome and metabolites

**DOI:** 10.1038/s41598-023-43964-4

**Published:** 2023-10-06

**Authors:** Dingnan Wang, Zhili Zheng, Han Yu, Dou Dou, Yining Gao, Shuang Xu, Zhiming Li, Lili Sun, Xudong Qiu, Xianggen Zhong

**Affiliations:** 1 Institute of Synopsis of Golden Chamber Department, School of Chinese Medicine College, Beijing University of Chinese Medicine, Beijing, 100029 People’s Republic of China; 2 Formulas of Chinese Medicine, Basic Medical College of Chengdu University of Traditional Chinese Medicine, Chengdu, 611137 Sichuan People’s Republic of China

**Keywords:** Immunology, Microbiology, Molecular biology, Biomarkers, Molecular medicine, Pathogenesis, Rheumatology, Risk factors

## Abstract

Studies have shown that high humidity is a condition that aggravates the pain of rheumatoid arthritis (RA), but the relevant mechanism is controversial. Currently, there is a lack of experimental animal studies on high humidity as an adverse factor related to the pathogenesis of RA. We used healthy SD rats and collagen-induced arthritis (CIA) rats to investigate the effects of high humidity on arthritis. Integrated metabolomics analyses of faeces and 16S rRNA sequencing of the faecal microbiota were performed to comprehensively assess the diversity of the faecal microbiota and metabolites in healthy and CIA rats. In this study, high humidity aggravated arthritis in CIA rats, which manifested as articular cartilage lesions, increased arthritis scores, and an increase in proinflammatory cytokines. High humidity had a certain effect on the articular cartilage extent, arthritis score and proinflammatory cytokines of healthy rats as well. Furthermore, high humidity caused significant changes in faecal microbes and metabolites in both healthy and CIA rats. 16S rRNA sequencing of faecal samples showed that high humidity increased the amount of inflammation-related bacteria in healthy and CIA rats. Faecal metabolomics results showed that high humidity significantly altered the level of faecal metabolites in healthy rats and CIA rats, and the changes in biological functions were mainly related to the inflammatory response and oxidative stress. Combined analysis showed that there was a strong correlation between the faecal microbiota and faecal metabolites. High humidity is an adverse factor for the onset and development of RA, and its mechanism is related to the inflammatory response and oxidative stress. However, the question of how high humidity impacts RA pathogenesis needs to be further investigated.

## Introduction

Rheumatoid arthritis (RA) is an autoimmune disease characterized by inflammatory changes in synovial tissues, cartilage, and bone^1^; chronic destructive polyarthritis is the main clinical manifestation of RA. The aetiology of RA remains unclear, and previous studies have shown that genetic and environmental factors can promote the development of RA^2,3^. Periodontal disease, smoking and diet can induce RA onset in genetically susceptible individuals^4–6^. It is also commonly reported that climate and the environment are associated RA pathogenic factors^7^.

Cold and humidity are climate and environmental factors associated with an increased risk for RA^8–11^. Studies have shown that weather can affect the pain of RA in middle-aged patients^12^, especially in female patients aged 41–65 years^13^. Low temperature, high air pressure and high humidity are significantly correlated with pain in RA patients^14^. Humidity is a frequently studied climate and environmental factor. An animal experiment showed that high humidity aggravated the severity of arthritis in CIA mice by upregulating xylitol and L-pyroglutamate expression^15^. The results confirmed clinical observations that high humidity could aggravate pain and stiffness in RA patients^16^. Research has shown that the combination of temperature and humidity creates a microclimate near the skin, and the humidity of the microclimate is affected by skin sweat glands on the water vapour surface. The microclimate could increase pain in RA patients by producing local vapour pressure. There is a positive correlation between humidity in the microclimate and RA-related pain^17^. Furthermore, researchers found that women living in damp houses had a higher risk of knee aches^18^, which may be linked to the autoimmune response that is increased based on relative humidity. In a clinical study from 1998 to 2001, humid conditions at a health centre directly induced rheumatic symptoms, including RA^19^.

The microbiome is critical to the balance of the human immune system and regulates a variety of functions as part of the human immune system^20,21^. The structure of healthy faecal microbiota can maintain immune balance and inhibit inflammatory responses. The microbiota significantly influences the development of joint lesions in inflammatory diseases, including RA and osteoarthritis^22,23^. Furthermore, dysregulated faecal microbes are associated with a variety of autoimmune diseases, including psoriasis, RA, and other immune system diseases^24,25^. The faecal microbiota and RA are closely related. On the one hand, dysbiosis of the faecal microbiota is common in RA patients^26,27^; on the other hand, regulating a faecal microbiota imbalance can effectively alleviate the occurrence and development of RA^28^. Clinical studies have shown that actinomycetes are bacteria that may directly induce RA^29^. Moreover, early biomarkers of RA found in the body’s blood or other body and tissue fluids can be used for the early diagnosis of RA patients and the prediction of treatment effects^30^. The metabolism of the host is regulated by its own genes and the gene composition of the symbiotic microbiota in the body; furthermore, metabolomics can efficiently screen biomarkers and comprehensively analyse the molecular mechanism underlying the health of the body^31^. The combination of microbiome and metabolome analyses can provide a comprehensive picture of host-microbiome interactions^32^.

In this study, to investigate the relationship between humidity and the onset and development of RA, healthy SD rats and collagen-induced arthritis (CIA) rats were exposed to high humidity (80 ± 5%). Foot and ankle histopathology and joint analysis were used to evaluate the effect of high humidity on the onset and development of arthritis in healthy and CIA rats. The abundance of faecal microorganisms was analysed by 16S rRNA high-throughput sequencing technology. Differentially activated metabolic pathways and differentially expressed metabolites were identified by untargeted metabolomics via gas chromatography‒mass spectrometry (GC–MS). This study aimed to (1) explore the effects of high humidity on the joints of healthy rats and CIA rats, (2) determine the composition and abundance within the faecal microbiota in healthy rats and CIA rats under high-humidity conditions, (3) measure the composition of faecal metabolites in healthy rats and CIA rats under high-humidity conditions, and 4) correlate faecal microbial diversity with GC–MS untargeted metabolomics results to explore the relationship between high humidity and the onset and development of RA. Furthermore, this study aimed to determine the potential biological impact of high humidity to provide novel insights into the impact of high humidity on RA.

## Results

### Effects of high humidity on arthritis symptoms and inflammation in healthy rats and CIA model rats

**Figure 1:**
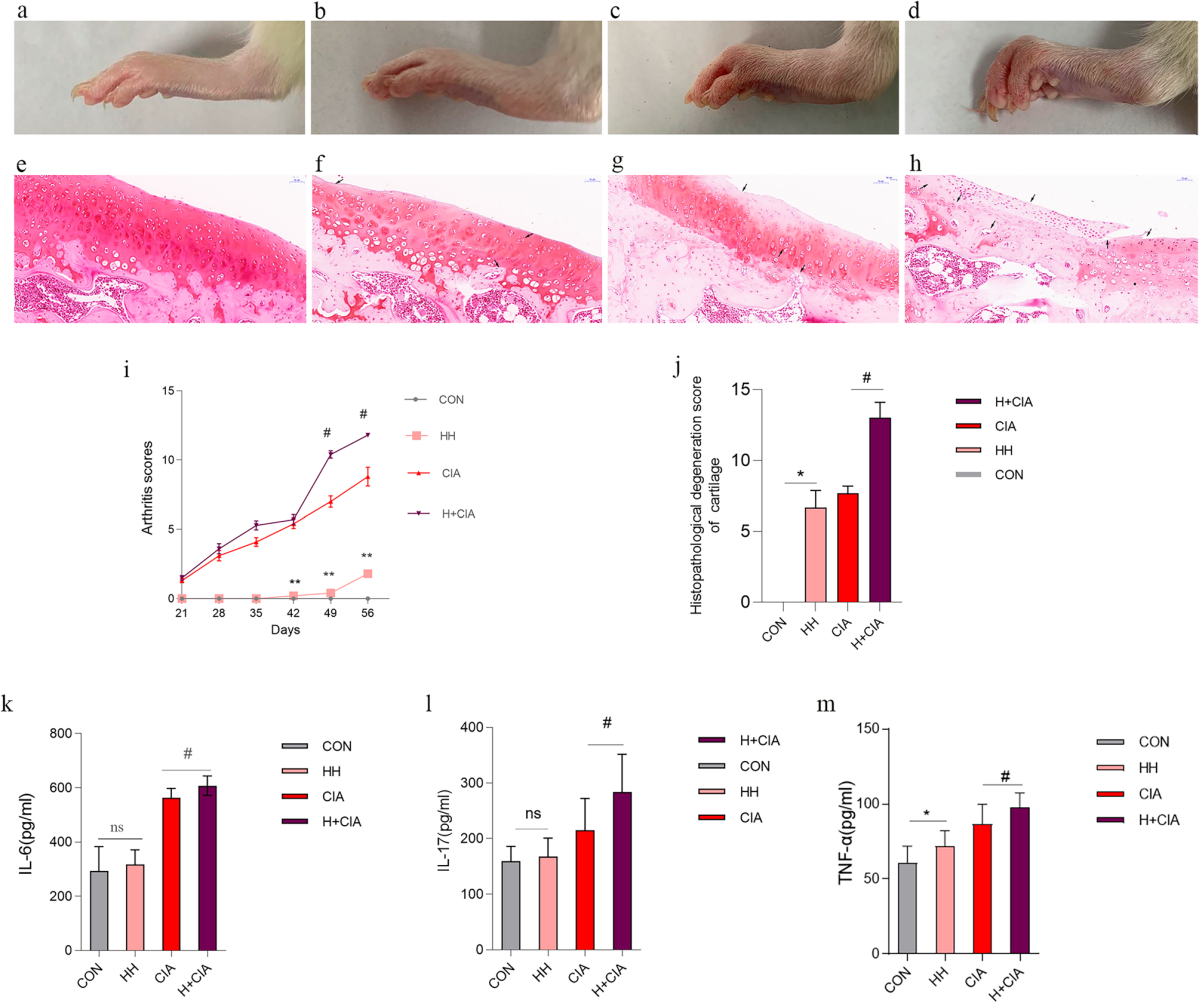
Effects of high humidity on arthritis symptoms and inflammation in healthy rats and CIA model rats. Representative images of ankle joints. (**a**) CON; (**b**) HH; (**c**) CIA; (**d**) H + CIA. Histopathological examination of articular cartilage by Saffron O staining. (**e**) CON; (**f**) HH; (**g**) CIA; (**h**) H + CIA. (**i**) arthritis scores. (**j**) Serum proinflammatory cytokines levels of rats in the each group. (**k**) IL-6; (**l**) IL-17; (**m**) TNF-α. Compared to that in the CON group, *p < 0.05, **p < 0.01; compared to that in the CIA group, ^#^*p*,0.05, ^##^*p* < 0.01; N.S, represents no significance.

To evaluate the effect of high humidity on arthritis, we observed changes in arthritis symptoms in healthy rats and CIA rats under normal-humidity (CON, 50 ± 5%) and high-humidity (HH, 80 ± 5%) conditions. Compared to those in the CON group (1a), ankle redness and swelling were observed in the HH group (1b). Compared to the CIA group (1c), the HH + CIA group exhibited more severe ankle swelling and joint deformation (1d). The effects of high humidity on articular cartilage in healthy rats and CIA rats are shown in Fig. 1. Compared to that in the CON group (1e), the staining of the cartilage in the HH group (1f.) was reduced. Compared to the CIA group (1 g), many of the cartilage samples were not stained, and chondrocytes were significantly reduced in the HH + CIA group (1 h). This result suggests that high humidity can cause or aggravate cartilage damage. Compared to those in the CIA group, arthritis scores were significantly increased in the HH + CIA group at Days 42, 49 and 56. Compared to those in the CON group, arthritis scores were significantly increased in the HH group at Days 49 and 56 (1i).

We investigated inflammation based on three proinflammatory cytokines in the rats of each group. Compared to the CON group, high humidity caused the upregulation of TNF-α in the HH group, and the difference was statistically significant (Fig. 1j). High humidity led to the upregulation of IL-6 and IL-17 in the HH group, but the difference was not statistically significant (Fig. 1k; 1 l). Compared with the CIA group, high humidity led to the upregulation of IL-6, IL-17 and TNF-α in the HH + CIA group (Fig. 1k; 1 l; 1 m), and the difference was statistically significant.

### Effects of high humidity on colonic symptoms and faecal moisture levels in healthy rats and CIA model rats

**Figure 2:**
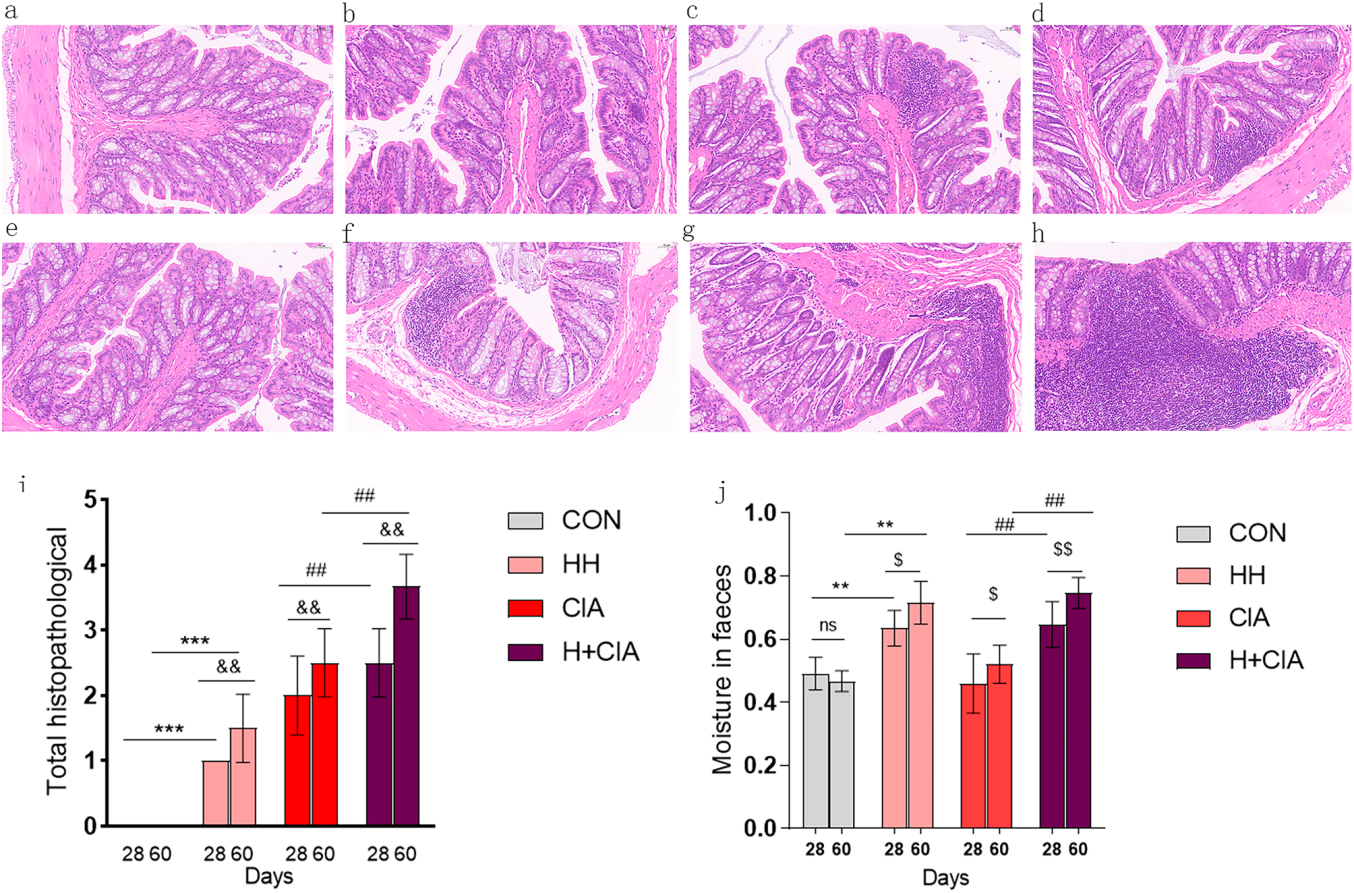
Effects of high humidity on colonic symptoms and faecal moisture levels were evaluated in healthy rats and CIA model rats. (**a**) CON 28 days; (**b**) HH 28 days; (**c**) CIA 28 days; (**d**) H + CIA 28 days; (**e**) CON 60 days; (**f**) HH 60 days; (**g**) CIA 60 days; (**h**) H + CIA 60 days. Histopathological examination of the colon by Hematein Eosin staining. (**i**) Histological scores. (**j**) Faecal moisture levels. Compared to that in the CON group, * *p* < 0.05, **p < 0.01; compared to that in the CIA group, ^#^*p*,0.05, ^##^*p* < 0.01; compared to that in the 60 days, ^$^*p* < 0.05, ^$$^*p* < 0.01, N.S, represents no significance.

We analyzed colonic pathology and fecal moisture levels on Day 28 and Day 60 to assess potential early changes in fecal characteristics. The impact of elevated humidity on the colon is depicted in Fig. 1. Notably, the HH group (Fig. 2b,f.) displayed enhanced accumulation of inflammatory cells compared to the CON group (Fig. 2a,e). Furthermore, compared to the CIA group (Fig. 2c,g), the H + CIA group (Fig. 2d,h) exhibited pronounced mucosal surface damage along with exacerbated accumulation of inflammatory cells. Notably, on Day 60, the severity of inflammation and tissue damage was considerably more prominent in the HH group, CIA group, and H + CIA (Fig. 2f,g,h) group compared to Day 28. The histological scores are presented in Fig. 2i.

Regarding faecal moisture content, a significant increase was observed in the HH group relative to the CON group. Similarly, the H + CIA group showed a significantly higher faecal moisture content than the CIA group (Fig. 2j). Of particular interest was a significant elevation in faecal moisture levels on Day 60 compared to Day 28 in the HH group, CIA group, and H + CIA group.

### Faecal Microbiota Analysis

**Figure 3:**
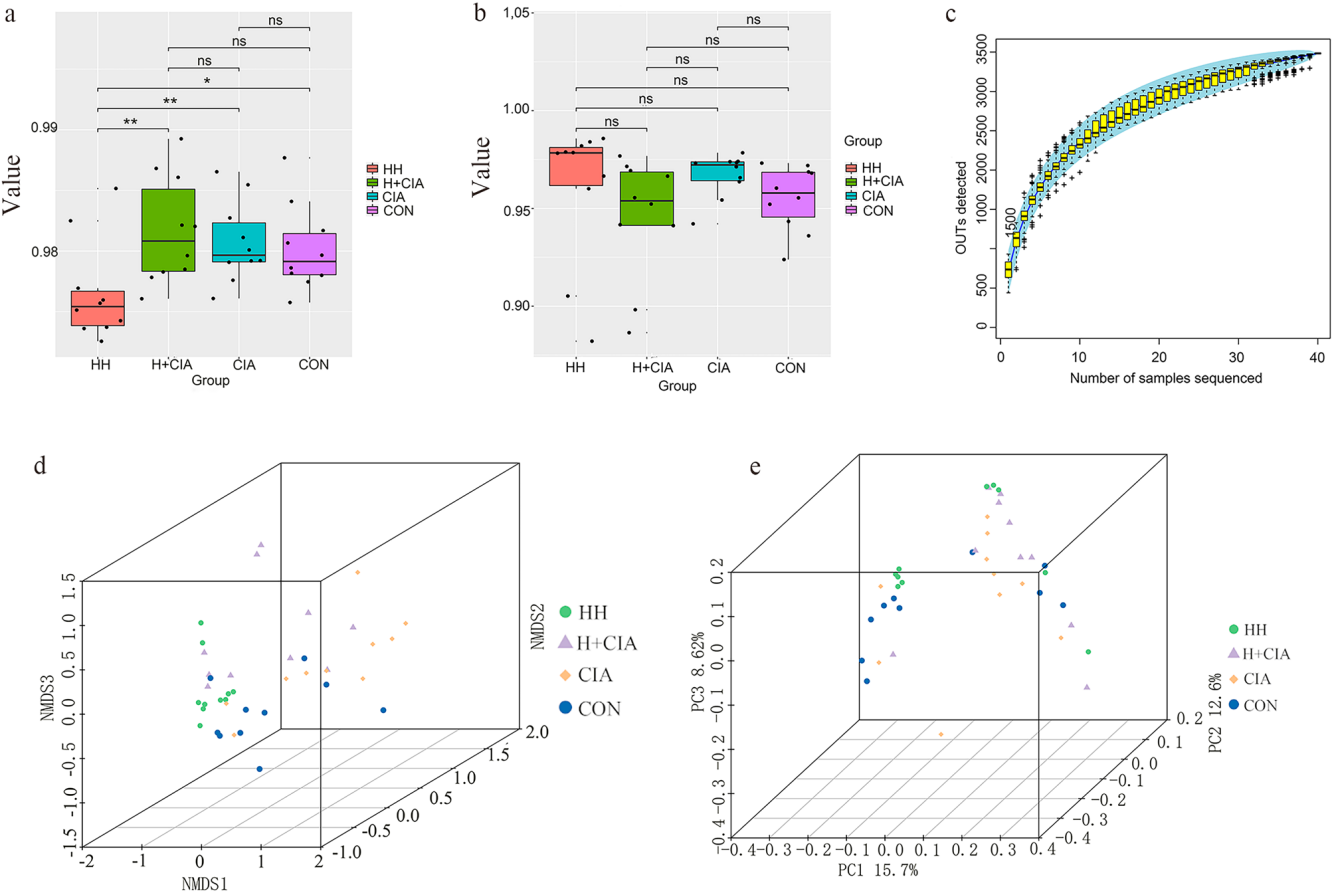
Species abundance and diversity. (**a**) Good’s diversity analysis; (**b**) Shannon’s diversity analysis; (**c**) Specaccum species accumulation curve; (**d**) NMDS analysis (Strees:0.08); (**e**) PCoA analysis (P = 0.0019).

Alpha diversity analysis usually reflects faecal microbiome abundance. Good’s diversity index (3a), Shannon’s diversity index (3b) and the Specaccum species accumulation curve (3c) are shown in Fig. 3. The results showed that the sequencing data fully reflected the information about bacterial communities in the samples. The results of beta diversity analysis reflected sample differences between groups. Principal coordinate analysis (PCoA, unweighted UniFrac, Fig. 3d) and nonmetric multidimensional scaling (NMDS) (Fig. 3e) were used to analyse the effects of humidity on the microbial communities of each group. The results showed that there were significant differences among the groups, and high humidity had significant effects on the microbial communities of healthy rats and CIA rats.

**Figure 4:**
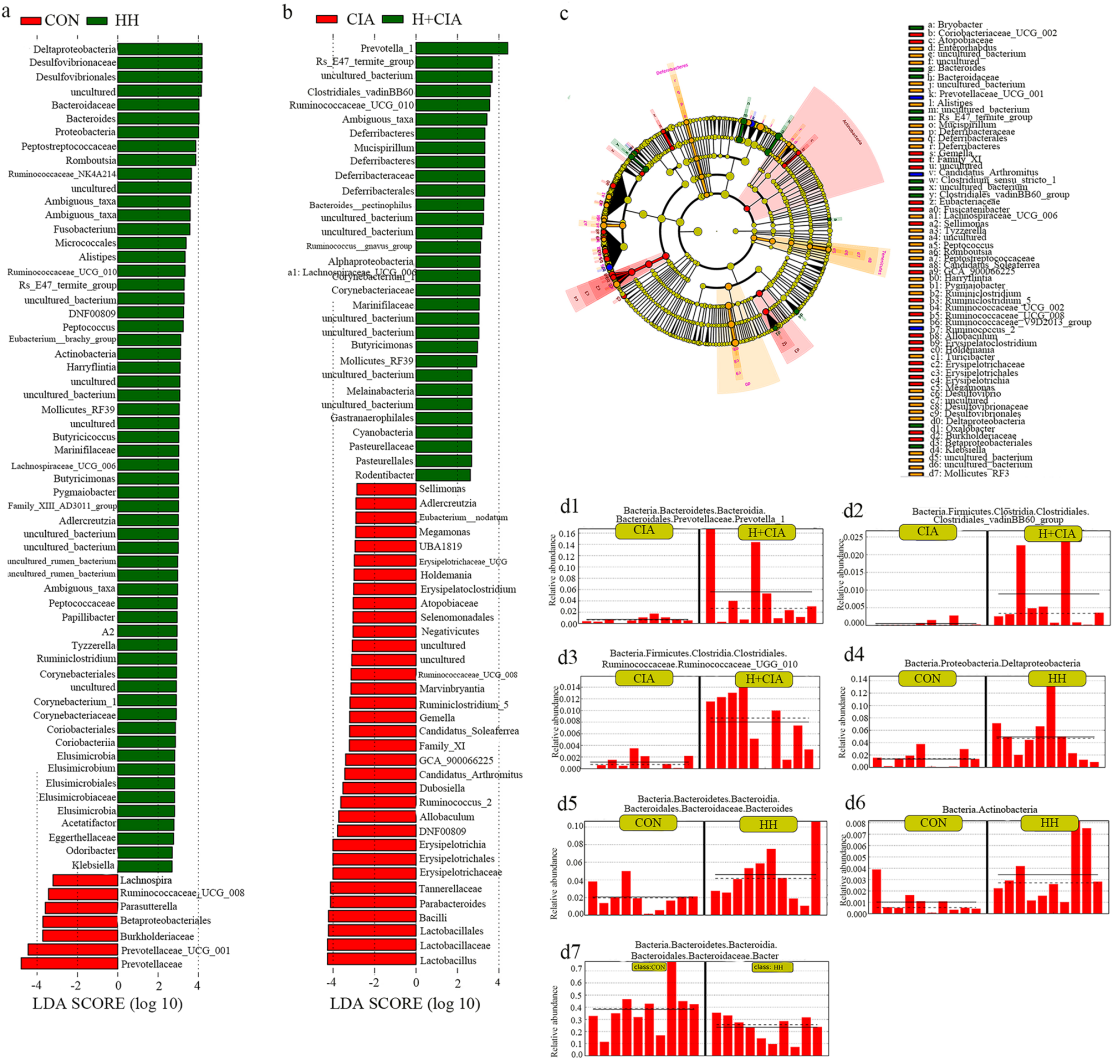
Statistical analysis of microbial multivariate. (**a**) Differential species score map of HH vs. CON groups; (**b**) differential species score map of H + CIA vs. CIA groups; (**c**) example diagram of annotated branches of different species; (**d**) histogram of relative abundance. The solid line is the mean value of the relative abundance, and the dashed line is the median value of relative abundance. CIA VS. H + CIA group: d1, Prevotella; d2, Clostridial; d3, Ruminococcaceae_UCG_010. CON VS. HH group: d4, Deltaproteobacteria; d5, Bacteroidaceae; d6, Actinobacteria; d7, Prevotella.

Then, linear discriminant analysis coupled with effect size measurement analysis (LEfSe) was used to identify biomarkers with significantly different abundances between each group (Fig. 4c). Compared to those in the CIA group, the most common bacteria in the HH + CIA group were Prevotella, Clostridial spore-forming bacteria and Ruminococcaceae_UCG_010 (Fig. 4b); compared to those in the CON group, the most common bacteria in the HH group were Deltaproteobacteria, Bacteroidaceae and Actinobacteria (Fig. 4a). Notably, compared with that in the CON group, the abundance of Prevotella species in the HH group was reduced. This information is collectively shown in Fig. 4d.

### Multivariate analysis of faecal metabolite profiles

**Figure 5:**
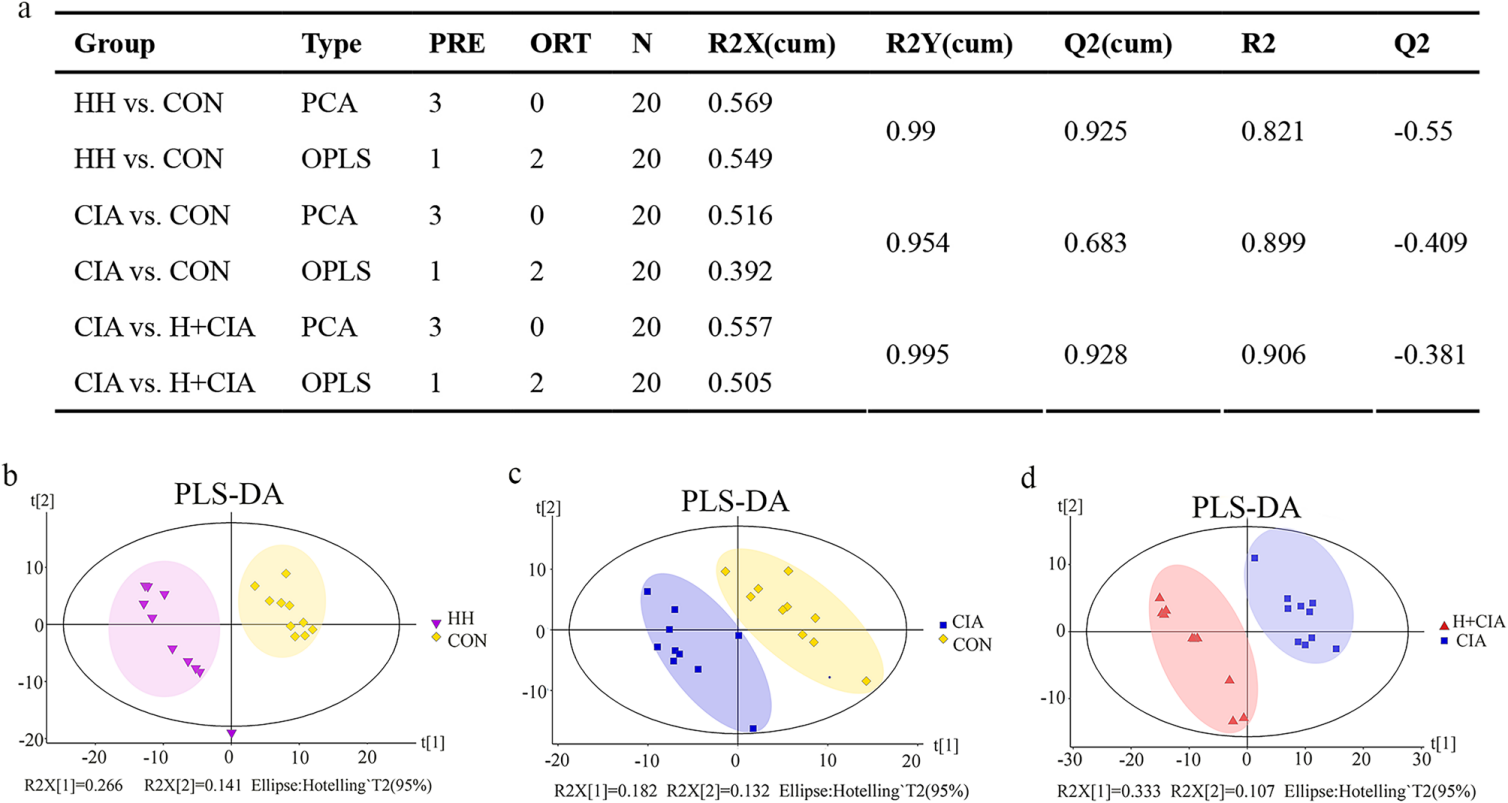
Multivariate statistical analysis of metabolic profiles in each group. (**a**) PCA parameters; (**b-d**) PLS-DA analysis results of each group.

The score chart of PLS-DA showed significant differences (spectrum separation) between each group, and the ratios of R2 X (CUM), R2 Y (CUM) and Q2 (CUM) are shown in Fig. 5a. PLS-DA was used to screen out differential variables (Fig. 5b-d).

**Figure 6:**
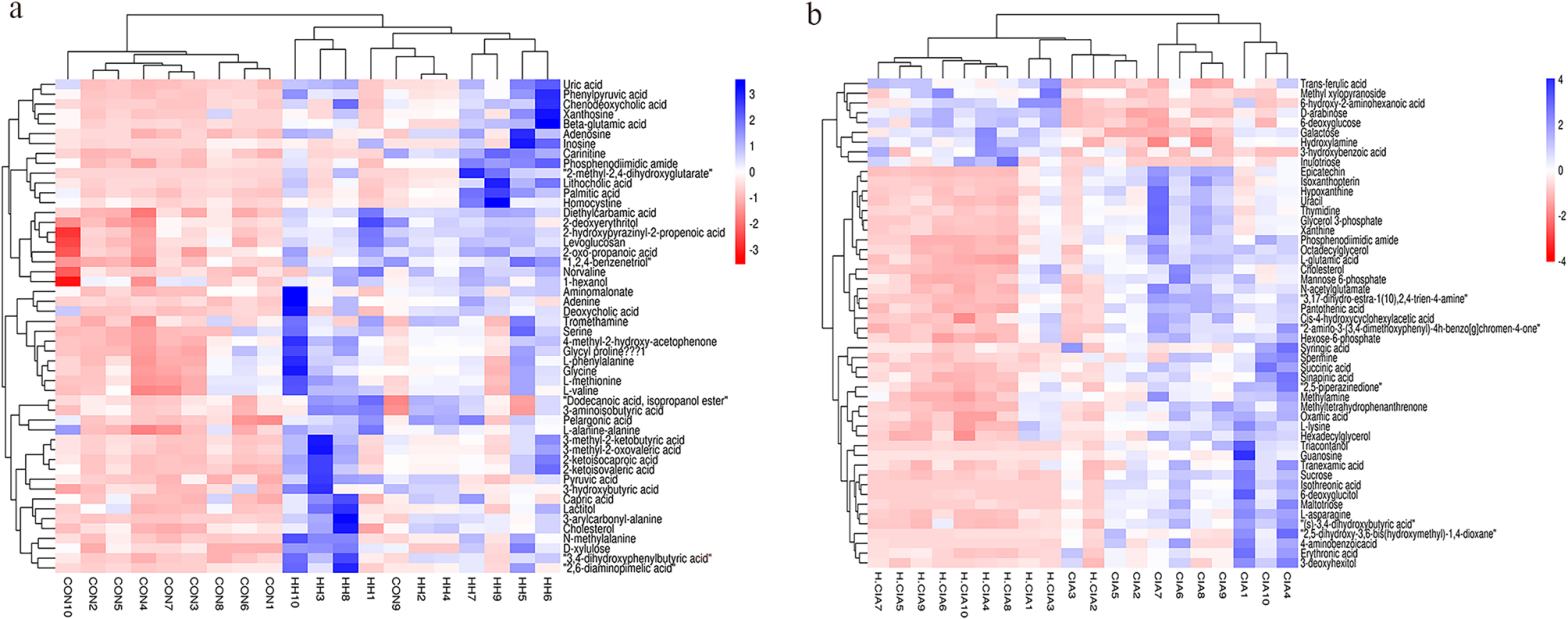
Heatmap of the differential metabolites. (**a, b**) Heatmap of HH vs. CON groups, H + CIA vs. CIA groups, respectively. Heatmaps were obtained from the cloud platform of Shanghai OE Biotech, Inc. ( https://cloud.oebiotech.cn/task/detail/heatmap/, version 1.26).

Differentially expressed metabolites were determined based on a VIP > 1 and an adjusted* p* value < 0.05. The results showed that a total of 108 metabolites were significantly expressed between the HH group and CON group, and 119 different metabolites were significantly differentially expressed in the HH + CIA group and CIA group. In the HH group vs. CON group comparison, the subclasses of differentially expressed metabolites were associated with amino acids, peptides and analogues (18 differentially expressed metabolites), carbohydrates and conjugates (8 differentially expressed metabolites), and fatty acids and conjugates (6 differentially expressed metabolites). In the HH + CIA group vs. CIA group comparison, the subclasses of differentially expressed metabolites were associated with organic acids and derivatives (24 differential metabolites), lipids and function-like molecules (18 differential metabolites), and organic oxygen compounds (16 differential metabolites). The top 50 differentially expressed metabolites between each group are presented as a heatmap in Fig. 6.

### Pathway analysis of differentially expressed metabolites

**Figure 7:**
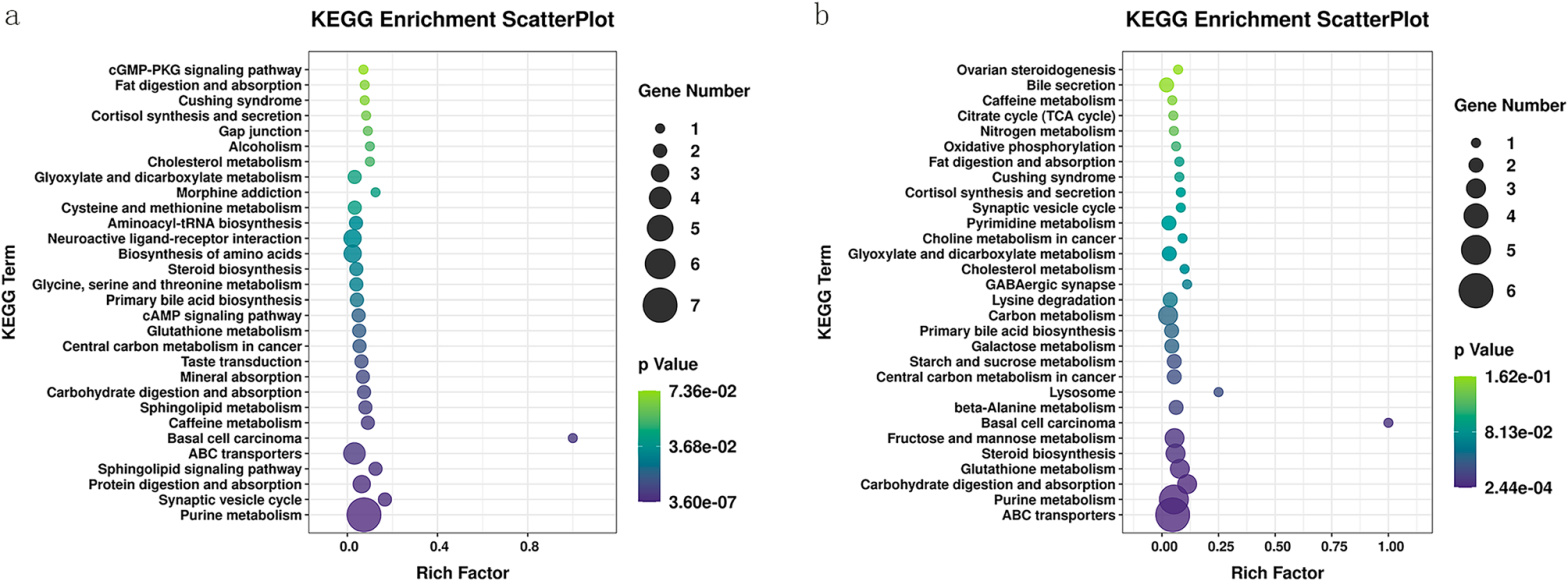
Scatterplot diagrams of KEGG pathways^33^. (**a**) HH vs. CON groups; (**b**) H + CIA vs. CIA groups. The x-axis shows the Rich factor, the colour of each circle indicates the *p* value, and the size of each circle reflects the number of metabolites of each pathway.

Kyoto Encyclopedia of Genes and Genomes (KEGG) analysis was used to further analyse the function of different metabolites, and the results of significant pathway enrichment analysis are presented on a scatterplot graph (Fig. 7). The following 8 pathways of the top 20 pathways were activated when comparing to the HH vs. CON groups and the HH + CIA vs. CIA groups: ABC transporter pathways, purine metabolism, carbohydrate digestion and absorption, glutathione metabolism, steroid biosynthesis, basal cell carcinoma pathways, central carbon metabolism pathways in cancer, and primary bile acid biosynthesis.

### Correlation of the faecal microbiota and metabolites

**Figure 8:**
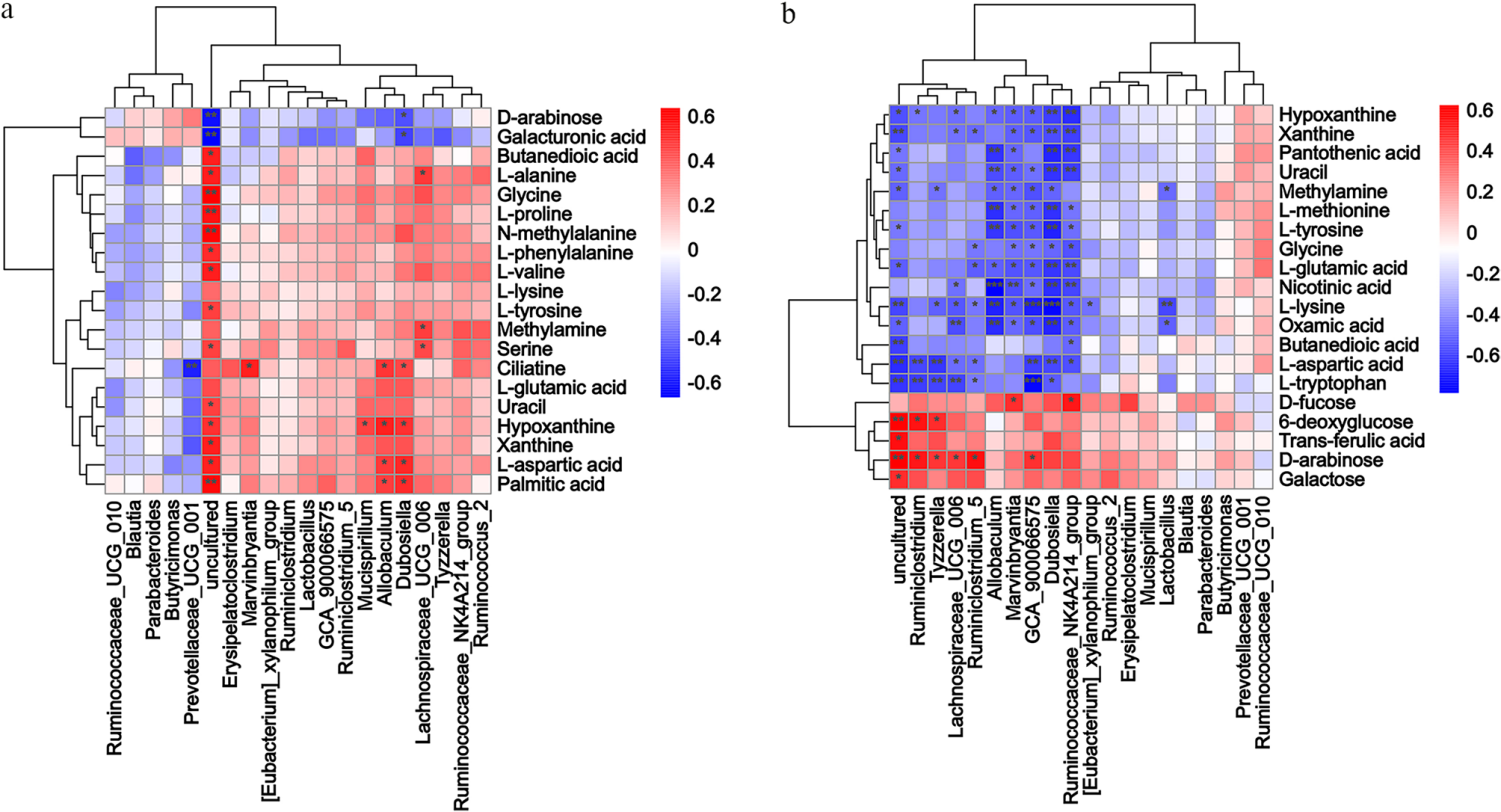
Correlation of the faecal microbiota and faecal metabolites. (**a**) HH vs. CON groups; (**b**) H + CIA vs. CIA groups. *p < 0.05, **p < 0.01. The correlation matrices were obtained from the cloud platform of Shanghai OE Biotech, Inc. (https://cloud.oebiotech.cn/task/detail/correlation-multiomics-oehw/, version 1.8).

To comprehensively analyse the relationship between faecal metabolism and the faecal microbiota, Spearman correlation analysis was performed for the HH vs. CON groups (Fig. 8a) and the HH + CIA vs. CIA groups (Fig. 8b). A correlation matrix network was constructed. The results showed that the paired correlations between the HH vs. CON groups and the HH + CIA vs. CIA groups indicated a strong correlation between the faecal microbiota and faecal metabolites. Prevotellaceae was identified as a significantly regulated microbial taxon at the genus level in the comparison between the HH and CON groups. It showed a significant negative correlation with Ciliatine. In the comparison between the CIA and H + CIA groups, Ruminococcaceae was found to be a significantly regulated microbial taxon. Specifically, within the Ruminococcaceae family, the Ruminococcaceae_NK4A214_group exhibited a positive correlation with D-fucose while displaying a negative correlation with 13 amino acids, including L-glutamic acid. Furthermore, the lactobacillus species, closely associated with RA, negatively correlated with L-lysine, oxamic acid, and methylamine.

## Discussion

Weather and environmental factors can directly impact human health. Relative humidity has been shown to play an important role in the study of climate sensitivity in patients with chronic pain. Studies have shown that high humidity is a direct or indirect factor affecting many diseases^34,35^. To date, there has been a lack of experimental studies regarding high humidity as an adverse factor associated with the pathogenesis of RA. In this study, healthy and CIA rats were exposed to high-humidity, and it was confirmed that high humidity can aggravate the extent of arthritis symptoms, including pathological changes, arthritis score and inflammatory factor expression. At the same time, the mechanism of the effect of high humidity on the joints of healthy rats and CIA rats was explored from the perspective of faecal microbial and metabolite homeostasis.

To account for these changes, faecal microbiome analysis was performed on the faeces of rats from each group. The faecal microbiota may indicate environmental risk factors for RA^36^. In this study, Prevotella, Clostridia, and Ruminococcaceae_UCG_010 were the most common bacteria after high humidity intervention in CIA rats, while Deltaproteobacteria, Bacteroidaceae, and Actinobacteria were the most common bacteria in high humidity intervention healthy rats. The faecal microbiome influences innate and adaptive immunity, and its imbalance can trigger inflammatory responses and increase the risk of autoimmune disease, leading to joint damage^37,38^. Animal experiments have shown that Prevotella, Ruminococcaceae and Clostridia were associated with RA because they induce the inflammatory response mediated by IL-6, IL-17 and IFN-γ cytokines^39^. In addition, studies have shown that Prevotella bacteria exhibit properties that increase inflammatory responses, possibly related to its ability to drive immune responses to Th17 cytokines^40^. In this study, high humidity aggravated joint injury in CIA rats, which may be related to the high abundance of bacterial species such as Prevotella, Clostridia, and Ruminococcaceae_UCG_010 induced by high humidity, which could promote the immune response of Th17 cells. It is possible that Th17 cell differentiation is activated and inflammatory cytokines increase, which would then induce an inflammatory response and aggravate joint injury. Notably, Deltaproteobacteria, Bacteroidaceae and Actinobacteria were significantly abundant in the HH group in this study. Previous studies have shown that Bacteroidaceae and Deltaproteobacteria are associated with RA^4,41,42^, while Actinobacteria, as a pathogen closely related to RA, may be an important factor that directly induces RA in damp environments^19,29^. At the same time, compared with the CON group, the abundance of Prevotellaceae in the HH group was significantly reduced, suggesting that high humidity intervention reduced the abundance of Prevotellaceae in healthy rats. Previous animal studies have shown that the abundance of Prevotellaceae decreased during the immune activation stage of CIA^43^, which is consistent with our results. We speculate that high humidity may cause a certain degree of immune response, which may be related to RA. However, the objectivity of this conclusion still needs to be further explored.

Combined studies on the microbiome and metabolome are considered to be one of the best ways to assess host-microbiome interactions^44^. The pathogenesis of RA is multifactorial, and oxidative stress and inflammatory responses are associated with the onset and development of RA. In this study, ABC transporter pathways, primary bile acid biosynthesis, and glutathione metabolism were found to be the metabolic pathways that were significantly enriched, and all of these are associated with oxidative stress^45^. Carbohydrate digestion and absorption and steroid biosynthesis were also metabolic pathways with significant enrichment, and these pathways are both related to digestion and absorption of the diet. Thus, we provide the following two explanations for these phenomena: 1. It has been confirmed in relevant reports^46^ and our previous reports^47,48^ that cholestasis may cause oxidative stress. ABC transporters play an important role in the pathogenesis of cholestasis, as they participate in the regulation of tryptophan metabolism and promote the synthesis of glutathione. Inhibition of ABC transporters could cause an inflammatory response, which would induce the production of reactive oxygen species (ROS). The content of mitochondrial ROS in monocytes of RA patients increases fivefold. Oxidative stress caused by ROS may be associated with the pathogenesis of RA. The onset and development of RA in the HH and HH + CIA groups may be closely related to oxidative stress. The mechanism by which high humidity interferes with ABC transporter pathways and primary bile acid biosynthesis could be related to ROS production and glutathione synthesis disorder, which are induced by the abnormal metabolism of bile acid. 2. Diet metabolism is closely related to the inflammatory process. Studies have shown that the abnormal metabolism of carbohydrates and fatty acids can promote inflammatory responses^49^. Disturbed carbohydrate absorption^50^ stimulates the inflammatory response by interfering with TNF-α and IL-6 production. Dysregulation of lipid metabolism homeostasis causes an immune reaction by increasing inflammatory factors such as TNF-α^51^. Therefore, high humidity may promote the inflammatory response by regulating metabolic pathways such as digestion and absorption, steroid biosynthesis and the biosynthesis of other metabolites, thus promoting the development of arthritis in CIA rats. In conclusion, high humidity can lead to the occurrence and development of RA by affecting oxidative stress and promoting the inflammatory response. Interestingly, these pathways also appeared in the comparison between the HH and CON groups. We speculated that high humidity may impact RA by inducing oxidative stress and promoting the inflammatory response; however, this result still needs further verification. In addition, Spearman correlation analysis was used to establish a correlation matrix to comprehensively analyse the relationship between faecal metabolites and the faecal microbiota. Our results suggest a potential interrelationship between faecal metabolites and faecal microbes.

In conclusion, the results of our study indicate that high humidity can impact the onset and development of RA, and the mechanism is likely related to the inflammatory response and oxidative stress. Nevertheless, it is crucial to acknowledge that our study represents a preliminary exploration, warranting further investigation into the impact of high humidity on the risk of RA in healthy rats. Next, we need to make experiments on how high humidity causes faecal microbiota changes and how these changes affect joint inflammation. In particular, the relationship between environmental humidity and the onset and development of RA can be further explored by extending the experimental observation period, adjusting the environmental humidity and verifying these results in clinical trials, cell and microorganism, and vitro and vivo mechanism validation experiments.

## Materials and methods

### Animals and husbandry details

Six-week-old healthy male SD rats were used in accordance with the Guidelines for the Care and Use of Laboratory Animals of the Institute of Laboratory Animal Resources, Institutional Animal Care and Use Committee of Beijing University of Chinese Medicine. The study is reported in accordance with ARRIVE guidelines. The animal weights were between 135 and 165 g at the beginning of the first study. All rats (n = 40) were housed with 5 animals per cage on a 12 h/12 h light/dark cycle in the animal facility of the Experimental Center of Beijing University of Chinese Medicine (room temperature: 25 ± 1 °C, humidity: 50 ± 5%). All animals were allowed ad libitum access to food and water.

### Treatment and sample collection

**Figure 9:**
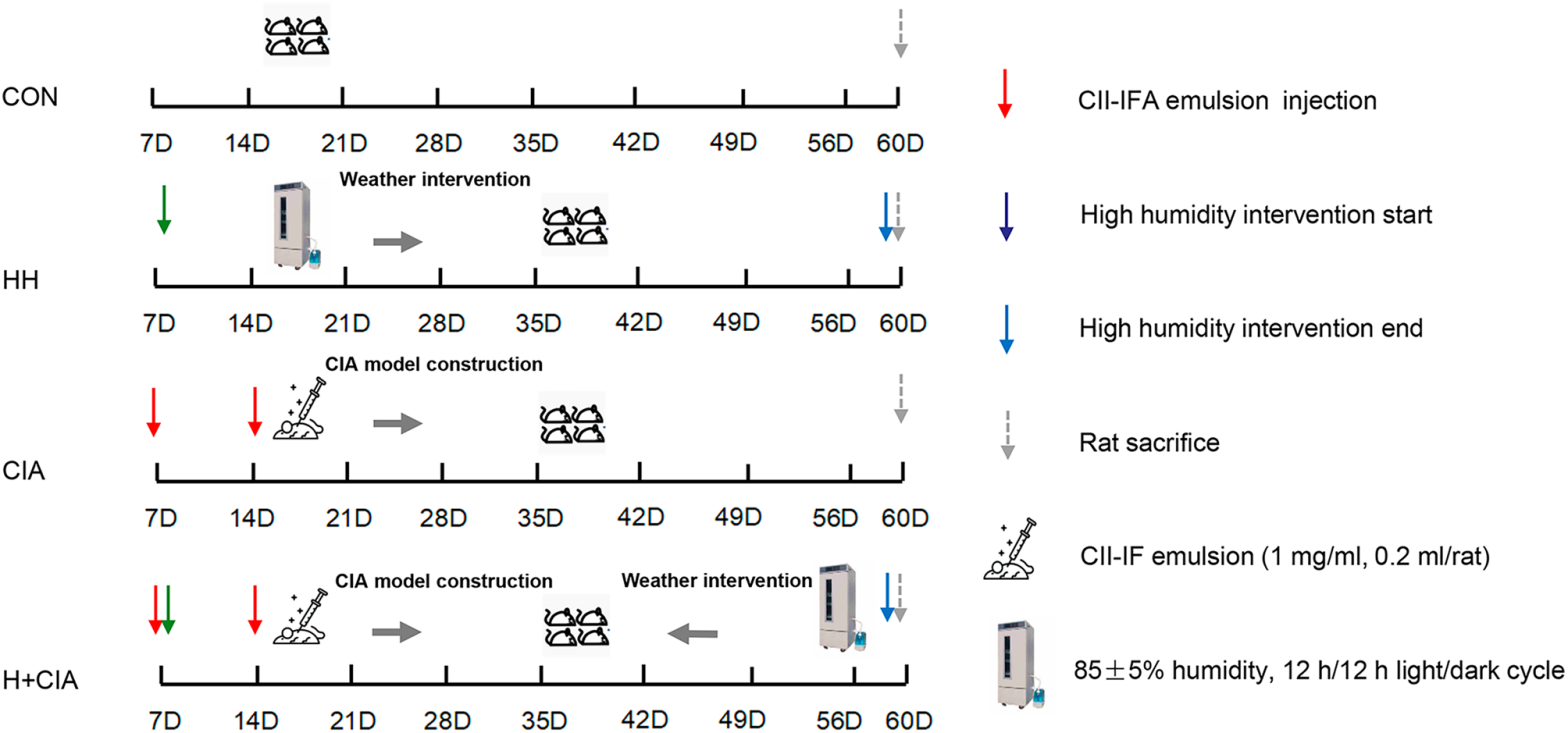
Group information, time course and experimental procedure. D, day. Isolators for maintaining a rat's humid climate provide a complete barrier around rat cages. The isolators allow for the adjustment of different humidity and temperature levels.

Bovine type II collagen (CII; 2 mg/ml) (Chondrex Inc., WA, United States) emulsified with complete Freund’s adjuvant (CFA; 2 mg/ml) (Sigma–Aldrich Co., St. Louis, United States) was obtained. All animals were divided into the following 4 groups (N = 20) with the random number method after acclimation for 1 week: ① control (CON) group, kept at 25 ± 1 °C, 50 ± 5% humidity and injected with 0.9% NaCl solution (0.2 ml/rat) on Days 7 and 14; ② high humidity (HH) group, kept at 25 ± 1 °C, 80 ± 5% humidity and injected with 0.9% NaCl solution (0.2 ml/rat) on Days 7 and 14; ③ CIA model (CIA) group, kept at 25 ± 1 °C, 50 ± 5% humidity and injected with CII-CFA emulsion (1 mg/ml, 0.2 ml/rat) on Days 7 and 14; and ④ CIA model with high humidity (HH + CIA) group, kept at 25 ± 1 °C, 80 ± 5% humidity and injected with CII-CFA emulsion (1 mg/ml, 0.2 ml/rat) on Days 7 and 14. Arthritis scores were measured every 7 days after Day 21^15,33^. Two fresh stool pellets from each rat were collected after Day 59. Faecal moisture levels were measured on Day 28 and Day 56. Samples were placed in sterile conical tubes and immediately frozen at −80 °C. Rats were anaesthetized by intraperitoneal injection of 2% sodium pentobarbital (0.2 ml/100 g). Ten rats from each group were randomly selected and euthanized after 28 days to isolate colonic tissues to observe histopathological changes. The remaining rats were euthanized and the hind limbs of the rats were collected on Day 60. Blood samples were collected from the inferior vena cava of rats and centrifuged at 3500×*g* and 4 °C for 15 min to collect the serum, which was immediately frozen at −80 °C. The group information and experimental process are shown in Fig. 8 are shown in Fig. 9.

### Assessment of arthritis variables

Arthritis scores were measured every 7 days after Day 21. The assessment criteria of arthritis scores were as follows: 0, no redness and swelling in the foot joints and claws; 1, mild swelling or redness of the foot joint and claws; 2, moderate swelling or mild redness of the foot joint and claws; 3, the claws are red and swollen below the ankle joint; and 4, severe redness, swelling and deformation of the ankle foot joint.

The articular cartilage was fixed in 4.0% paraformaldehyde for 3 days and then decalcified in 10% ethylenediamine tetraacetic acid (EDTA) decalcification solution. The tissues were dehydrated by an ethanol gradient, embedded in paraffin, and sliced into 4 μm sections by pathological sectioning. Then, the slides were stained with safranin O to assess articular cartilage damage.

### Measurements of serum proinflammatory cytokines

The levels of TNF-α, IL-6 and IL-17 in serum were measured by enzyme-linked immunosorbent assay (ELISA). All reagents were purchased from CUSABIO (CUSABIO Technology LLC, Wuhan, China), and analyses were conducted according to the manufacturer’s instructions. The absorbance was measured at 450 nm.

### DNA extraction and 16S rRNA sequencing

According to the manufacturer's instructions, a DNeasy PowerSoil kit (Cat. No. 12888; QIAGEN, Dusseldorf, Germany) was utilized to extract DNA from different samples. The purity and concentration of DNA were detected by agarose gel electrophoresis. Extracted DNA was diluted to a concentration of 1 ng/μl and stored at −20 °C for further processing. The DNA genome was utilized as a template for PCR amplification to ensure the efficiency and accuracy of amplification with barcode-specific primers and Takara Ex Taq hi-fi enzyme (Cat. No. RR001Q; Takara, Dalian, China). The corresponding areas of bacterial diversity identification were as follows: The V3-V4 region of the 16S rRNA genes was amplified with universally primed 343F (5' -TACgGRaggCAGCAGG-3') and 798R (5' -AgggTATCtaatCCT-3') using a commercial PCR kit (Cat. No. 51531; Qiagen, Dusseldorf, Germany).

PCR products were detected by gel electrophoresis and purified by AMPure XP beads after detection. The purified products were used as second round PCR templates and amplified by a second round of PCR. After a second purification step with AMPure XP beads, PCR products were quantitatively analysed by a Qubit dsDNA detection kit (Cat. No. Q32854; Thermo Fisher Scientific, MA, United States). The samples were mixed in equal quantities according to the concentration of PCR products and then sequenced. An equal amount of purified amplicon was pooled for sequencing with a NovaSeq PE250 instrument.

### Operational taxonomic unit (OTU) clustering and species annotation

Raw sequencing data were in FASTQ format. Paired-end reads were then pre-processed using Trimmomatic software (version 0.35) to detect and cut off ambiguous bases (N). The sliding window method was used to evaluate the average base quality. If the average mass value in the window was lower than 20, the back-end base was cut from the window. Paired-end reads were assembled using FLASH software (version 1.2.11). The stitching parameters were as follows: the smallest overlap was 10 bp, the largest overlap was 200 bp, and the maximum error matching rate was 20%. Sequences were further filtered as follows: sequences containing ambiguity were removed and reads with 75% of bases above Q20 were retained. Moreover, Quantitative Insights into Microbial Ecology (QIIME, version 1.8) was used to detect and remove chimeric sequences.

After the sequencing data were pre-processed to generate high-quality sequences, Vsearch software (Version 2.4.2) was used to classify the sequences into multiple operational taxonomic units (OTUs) according to their similarity. A parameter sequence similarity greater than or equal to 97% was classified as an OTU unit. The QIIME software package was used to select representative sequences of each OTU, and all representative sequences were annotated and blasted against the Unite database (ITS rDNA) using pynast (v0.1).

### Sample preparation and GC–MS analysis

The samples stored at −80 °C were thawed on ice, 60 mg of stool sample was accurately weighed and placed into a 1.5-ml centrifugation tube, 40 μl of internal standard (l-2-chloro-phenylalanine, 0.3 mg/ml, methanol configuration) was added to each sample, and 2 small steel balls and 360 μl of cold methanol were successively added. Samples were stored at −20 °C for 2 min and ground in a grinding machine (60 Hz, 2 min). Samples were sonicated in an ice water bath for 30 min, 200 μl of chloroform was added, and the mixture was vortexed (60 Hz, 2 min). Then, 400 μl of water was added, and the mixture was vortexed (60 Hz, 2 min). Ultrasonic extraction was performed in an ice water bath for 30 min, and the samples were allowed to stand at −20 °C for 30 min. Then, the extract was centrifuged for 10 min (13,000×*g*, at 4 °C), and 300 μl of the supernatant was put into a glass-derived bottle and dried in a centrifugal freeze dryer. Next, 80 μl of methoxamine hydrochloride pyridine solution (15 mg/ml) was added to each sample, followed by vortexing for 2 min and ice water ultrasonic treatment for 3 min. The oxime reaction was carried out for 90 min in an incubating shaker at 37 °C. A total of 80 μl of trifluoroacetamide (containing 1% chlorotrimethylsilane) derivatizing reagent and 20 μl n-hexane were added, and 11 internal standards (C8/C9/C10/C12/C14/C16, 0.8 mg/mL; C18/C20/C22/C24/C26, 0.4 mg/ml, all prepared in chloroform) were added at a volume of 10 μl, followed by vortexing for 2 min and reaction at 70 °C for 60 min. After the samples were removed, they were placed at room temperature for 30 min for GC–MS metabolomics analyses. All extraction reagents were precooled at −20 °C before use.

### Meteorological chromatography-mass spectrometric conditions

Metabolite analysis was carried out by a gas chromatograph-mass spectrometer (7890B-5977A; Agilent J&W Scientific, Folsom, CA, United States). Separation was performed by loading a 30-m × 0.25-mm × 0.25-μm DB-5MS fused silica capillary column (Agilent J&W Scientific, Folsom, CA, United States). The flow rate of the carrier gas, high-purity helium (purity not less than 99.999%), was 1.0 ml/min, and the inlet temperature was 260 °C. The injection volume was 1 μl, and the solvent delay was 5 min. The temperature programme was achieved using the following gradient: The initial temperature of the GC oven was 60 °C, and then the temperature was ramped to 125 °C at 8 °C/min, to 210 °C at 5 °C/min, to 270 °C at 10 °C/min, and to 305 °C at 20 °C/min and held steady for 5 min. Mass spectrometric conditions were as follows: ionization source, electron impact ionization; ion source temperature, 230 °C; quadrupole temperature, 150 °C; collision energy, 70 eV; solvent delay, 3 min; scan mode, full scan (scan mode); and mass scan range, m/z 50–500.

### Data pre-processing and statistical analysis

Raw GC–MS mass spectra were converted to ABF format files by Analysis Base File Converter software (version 4.0). Then, the data were imported into MS-DIAL software (version 3.9) for pre-processing. Finally, the raw data matrix including the sample information, the name of each peak, retention time, mass-to-charge ratio, and mass spectral response intensity (peak area) were derived. The NIST database (https://webbook.nist.gov/chemistry/) was used for material qualitative analysis. Principal component analysis (PCA) and partial least squares discriminant analysis (PLS-DA) were performed to visualize the changes in metabolites between the experimental groups after mean centering (Ctr) and Pareto variance (Par) scaling, respectively. Variable importance in the projection (VIP) values were calculated according to the PLS-DA model. A VIP > 1 was used to identify potential biomarkers. The PLS-DA model was tested for 200 response sequencing tests; the x-matrix was fixed, the variables of the previously defined classification Y matrix (such as 0 or 1) were randomly arranged n times (n = 200), and the corresponding PLS-DA model was established to obtain R2 and Q2 values of the random model. Linear regression was performed with R2Y and Q2Y of the original model, and the intercept values of the regression line and Y-axis were R2 and Q2, respectively, which were used to measure whether the model was overfitting. The VIP values were calculated based on the PLS-DA model, and *p* values were derived from a two-tailed Student’s t test using the normalized peak areas. The criteria for screening differentially expressed metabolites were VIP > 1 and *p* < 0.05.

### Statistical analysis

Student’s t test for unpaired data (95% confidence interval) was used for comparisons between each group using GraphPad Prism (Version 9.3; GraphPad Software, San Diego, CA, USA). The data are expressed as the mean ± standard deviation of the mean (S.D.). One-way analysis of variance (ANOVA) was used for measurement data of multiple groups, and least-significant difference (LSD) was used for pial comparison between groups. If homogeneity of variance was not satisfied, the rank sum test was used for comparison of multiple independent samples. Error bars represent the standard deviation. The degree of significance is indicated as *p < 0.05, **p < 0.01, ^#^*p* < 0.05 and ^##^*p* < 0.01.

### Ethics approval and consent to participate

The animal study was reviewed and approved by Ethics Committee of Beijing University of Traditional Chinese Medicine (approved animal experimental protocol number, BUCM-4-2020092905-3119).

## Data Availability

The datasets presented in this study can be found in online repositories. The names of the repository/repositories and accession number(s) can be found below: The 16 s rRNA dataset presented in this study has been deposited at https://www.ncbi.nlm.nih.gov/bioproject/PRJNA823862; Metabolights [accession: MTBLS4649].
